# Wandering Spleen in a Patient With Significant Medical History

**DOI:** 10.7759/cureus.35543

**Published:** 2023-02-27

**Authors:** John French, Cindy L Austin, Fola E Sodade, Zachary T Beam

**Affiliations:** 1 Department of Medicine, University of Missouri, Columbia, USA; 2 Department of Trauma Research, Mercy Hospital, Springfield, USA; 3 Department of General and Trauma Surgery, Mercy Hospital, Springfield, USA

**Keywords:** vacterl, wandering spleen, whirl sign, vacterl association, post-splenectomy vaccines, malone antegrade continence enema (mace), appendicostomy

## Abstract

The clinical presentation of a wandering spleen is characterized mainly by unspecific acute symptoms, ranging from diffuse abdominal pain to left upper/lower quadrant and referred shoulder pain to asymptomatic. This has challenged accelerated medical care and impeded the acquisition of confirmatory diagnosis; therefore, increasing morbidity and mortality risks. Splenectomy is an established operative procedure for a wandering spleen. However, there has not been enough literature emphasizing the clinical history of congenital malformations and surgical corrections as inferential tools for facilitating a decisive and informed procedure.

The case presented is of a 22-year-old female who reported to the emergency department with a five-day persistent left upper quadrant and left lower quadrant (LLQ) abdominal pain, associated with nausea. According to the medical history, the patient had a significant history of vertebral defects, anal atresia, cardiac anomalies, tracheoesophageal fistula, renal anomalies, and limb abnormalities (VACTERL) associated with congenital anomalies. By the age of eight years, the patient had undergone multiple surgical interventions, including tetralogy of Fallot repair, an imperforate anal repair with rectal pull-through, Malone antegrade continence enema (MACE), and bowel vaginoplasty. Computed tomography imaging of the abdomen revealed evidence of a wandering spleen in the LLQ with associated torsion of the splenic vasculature (whirl sign). Intra-operatively, appendicostomy was identified extending from the cecum in a near mid-line position, to the umbilicus, and carefully incised distally, preventing injury to the appendicostomy. The spleen was identified in the pelvis, and the individual vessels were clamped, divided, and ligated. Blood loss was minimal with no post-operative complications. This rare case report adds valuable teaching points about the treatment of wandering spleen in individuals with VACTERL anomalies.

## Introduction

Wandering spleen is a cause of acute surgical abdomen that can result in deleterious consequences if not identified or treated expeditiously. Often the challenge of accelerated medical care is increased by a delayed diagnosis due to the non-specific clinical presentation [1]. Symptoms can range from asymptomatic in some patients to diffuse abdominal pain [1]. Other symptoms can include Kehr's sign, which occurs when left upper quadrant (LUQ) pain is referred to the left shoulder pain [1]. Vague symptoms can result from the stalled acquisition of confirmatory diagnostic imaging, causing increased risks of morbidity and mortality [2].

Anatomically, the spleen is tethered to the stomach by the gastrosplenic ligament and to the left kidney by the splenorenal ligament. The spleen also is tethered between the spleen and left colic flexure by the splenocolic ligament and between the spleen and diaphragm by the phrenicosplenic ligament. Congenital anomalies or previous surgical interventions can distort the normal anatomic position of the spleen, leading to the spleen wandering within the abdomen. Radiologists have termed the whirled appearance of vessel torsion as a “whirl sign,” first described in 2003 as a description on computed tomography (CT) for the finding of a midgut volvulus [3]. For the diagnosis of wandering spleen, a CT change will display a whirled appearance of hyperdense, non-enhancing splenic vessels [4].

Vertebral defects, anal atresia, cardiac defects, tracheoesophageal fistula, renal anomalies, and limb abnormalities (VACTERL) association is classified as a non-random association of birth defects that affect multiple parts of the body. The typical findings are vertebral segmentation defects, anal atresia, tracheoesophageal fistula, cardiac defects, esophageal atresia, single umbilical artery, and radial and renal defects [5]. The association is the result of a defective mesodermal development during embryogenesis, typically prior to 35 days of gestational age [6,7]. VACTERL association occurs sporadically, without any specific, consistent, genetic abnormality identified. Sporadic cases have been associated with mutations in FGF8, HOXD13, ZIC3, PTEN, FANCB, FOXF1, and TRAP1 genes and mitochondrial DNA [8-12]. VACTERL association is given the term "association" because it includes a series of specific features that have been found to occur together more often than happening alone due to chance. VACTERL association occurs sporadically, although recurrence within a sibship may occur; the risk for recurrence in a sibling or a child is usually around 1% [7].

In this case, the authors describe the importance of recognizing anatomical variations associated with VACTERL congenital anomalies and demonstrate how previous surgical interventions can contribute to the complexity of the case. This article was previously presented as a meeting poster abstract at the 2022 Annual Mercy Research Colloquium on April 28, 2022.

## Case presentation

This 22-year-old female presented to the emergency department for evaluation of LUQ and left lower quadrant (LLQ) abdominal pain. The patient experienced approximately five days of unrelenting abdominal pain, along with associated nausea. She rated her colicky pain as 6-7 out of 10 and endorsed worsening pain with movement or lying on her left side. No fever or chills were present.

The patient presented with an extensive medical history significant for the following: tetralogy of Fallot repair, repair of imperforate anus with rectal pull-through, and Malone antegrade continence enema (MACE). The patient was catheterized of her umbilicus with antegrade continence enemas for stool continence. The patient has a complicated urological history going back to when she was a young child. Bladder augmentation, urethroplasty, and bowel vaginoplasty occurred at the age of eight. The patient has a congenitally absent left kidney. She also reported self-catheterization for neurogenic bladder.

**Figure 1:**
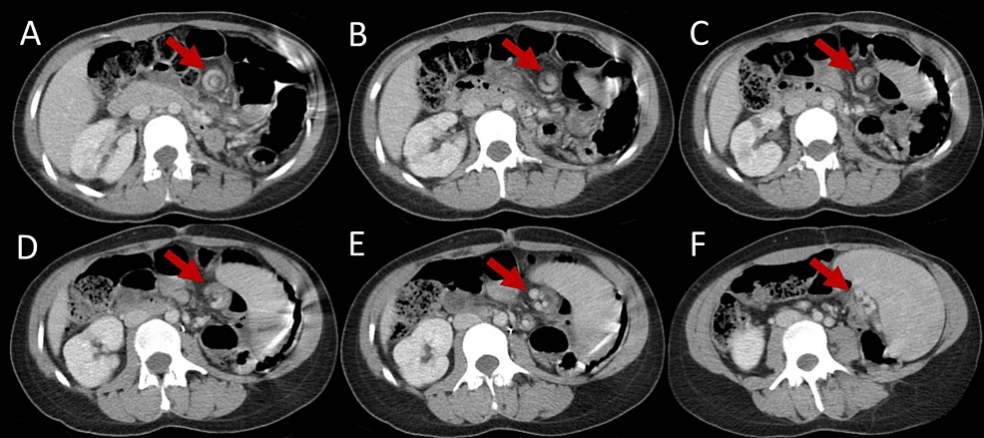
The "Whirl sign" from CT is depicted by red arrows. Images A-F show the progression of transverse CT acquisitions.

On examination, the patient was hemodynamically stable. She was afebrile. Her abdominal exam revealed tenderness to palpation in the LUQ of the abdomen without rebound tenderness, abdominal guarding, or rigidity. There was a palpable mass in the LLQ of the abdomen that was tender to palpation. Laboratory studies (complete blood count and comprehensive metabolic panel) were within normal limits. The CT imaging revealed evidence of a wandering spleen in the LLQ of the abdomen associated with torsion of the splenic vasculature (whirl sign) (Figure 1). The splenic vasculature was opacified, indicating perfusion, despite the significant torsion visualized on imaging. Since the patient had been lost to follow-up for more than 10 years regarding her cardiac issues, cardiology services were consulted to provide evaluation and opinion on cardiac risk with abdominal surgery. She underwent prompt cardiac evaluation. After reviewing cardiac diagnostic study results, she was deemed medically optimized from a cardiac perspective with moderate risk and proceeded to the operative theater for surgical intervention.

**Figure 2:**
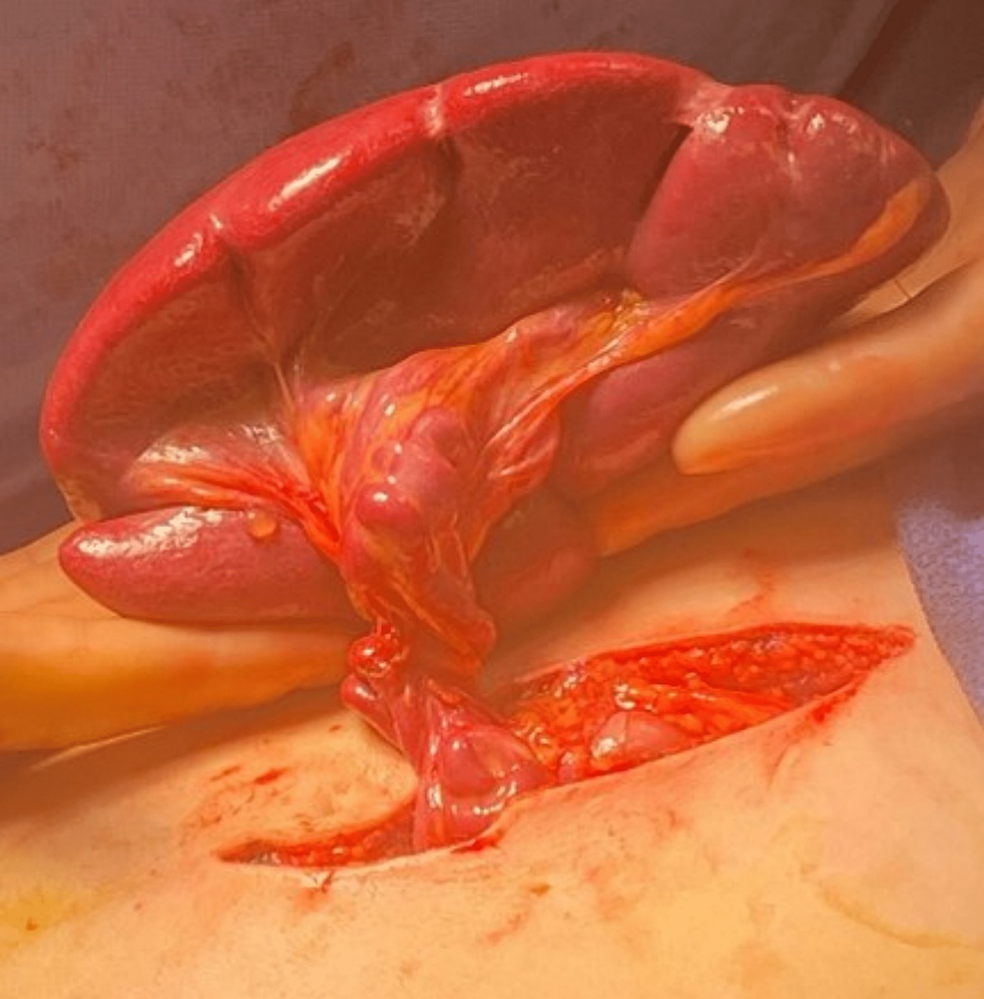
Torsion of the enlarged spleen with vascular torsion.

After informed consent was obtained, the patient was transported to the operating room and administered general anesthesia. An upper midline incision was carried through the subcutaneous fat and midline fascia using cautery. An appendicostomy was identified extending from the cecum, which was in a near midline position, to the umbilicus. The fascial incision extended distal to the umbilicus to avoid injury to the appendicostomy. The spleen was identified in the pelvis and removed through the upper midline incision (Figure 2). The splenic vessels were individually clamped, divided, and ligated. Then the appendicostomy was intubated with a red rubber catheter. The fascia was closed above and below the appendicostomy. The rubber catheter was withdrawn after the fascial closure. Blood loss was minimal with no complications in the post-anesthesia care unit. Post-splenectomy vaccines included *Haemophilus influenzae* type B, pneumococcal 13-valent conjugated, and meningococcal B. Postoperatively, the pain was controlled with intravenous therapy and oral medications. The patient remained in the hospital for an additional two days prior to discharge.

## Discussion

This case demonstrates the importance of obtaining a thorough history of past medical interventions. Without a prior history of VACTERL association congenital anomalies and surgical corrections, the patient could have been at a greater risk for intra- and postoperative complications. The reason being previous intra-abdominal surgery could have potentially caused the disruption of ligaments stabilizing splenic position; in addition to an absent left splenorenal ligament. Surgically, this case supports the importance of sufficiently taking time to identify, mark, and protect fragile anatomical structures. In this case, the patient had an appendicostomy structure at her midline that could have easily been overlooked and damaged with a midline incision. Resection or strangulation (from closing sutures) of her appendicostomy conduit would have led to a greater postoperative complication with the ability to induce bowel movements.

As described earlier, the wandering spleen can present as non-specific abdominal pain and is often a delayed diagnosis due to the vague clinical presentation. As a result, confirmatory imaging studies are stalled leading to increased morbidity and mortality. Thomas and Thomas (2021) described two cases of a wandering spleen with acute splenic torsion in the setting of an absent left kidney causing acute surgical abdomen with serious consequences [2]. Similar to this case, the patient had an absence of a left kidney resulting in loss of the splenorenal ligament. The splenorenal ligament is a key ligament necessary to maintain a normal splenic position in the abdomen. Furthermore, this case demonstrates how an acquired or congenital absent left kidney is associated with a wandering spleen as a result of an absence or weakness of the supporting suspensory splenic ligaments.

An important question that arises from this case is whether prophylactic spleen removal from a patient with VACTERL congenital anomalies is warranted. Patients with absent, acquired, or iatrogenic loss of their splenic ligaments, especially splenorenal and gastrosplenic ligaments, predispose to a possible necessity for emergent surgical intervention in the future. While prophylactic removal of the spleen could prevent the need for emergent surgical intervention, asplenia would cause reduced hematologic filtering and immunologic responses to pathogens. While additional research regarding this topic could pose some clinical and ethical challenges, ultimately, further investigation is needed to better characterize whether the risks would outweigh the benefits.

## Conclusions

This rare case adds valuable teaching points to the literature on radiological and preventative health maintenance. Radiologically, this case provides an appreciable illustration of the “whirl sign” that is associated with the torsion of splenic vasculature. In addition, this case supports the precedence of preventative health maintenance in patients with asplenia. Splenopexy should be considered unless significant torsion and/or infarction is identified. A thorough medical and surgical history should be obtained to ensure previous surgical interventions in infancy/childhood are identified and spared, especially in an emergent situation.
